# Diagnostic Challenges of an Abdominal Pregnancy in the Second Trimester

**DOI:** 10.1155/2021/7887213

**Published:** 2021-08-03

**Authors:** Niccole Ranaei-Zamani, Tetyana Palamarchuk, Swena Kapoor, Mandeep K. Kaler, Favour Atueyi, Rebecca Allen

**Affiliations:** ^1^The Royal London Hospital, Barts Health London, UK; ^2^Whipps Cross University Hospital, Barts Health, London, UK

## Abstract

Abdominal pregnancies are a rare form of ectopic pregnancy, which presents a significant risk of maternal morbidity and mortality. We describe an unusual case of a late diagnosis of an abdominal pregnancy in the second trimester, which due to diagnostic challenges, was not detected on 1st trimester and subsequent antenatal ultrasound scans (USS). The abdominal pregnancy was later diagnosed at the repeat anomaly scan and confirmed with a pelvic MRI. This case of abdominal pregnancy is unique when compared to other reported cases, as the fetus was initially enclosed within the amniotic sac with normal liquor volume. Both transvaginal and transabdominal scans appeared to demonstrate an intrauterine pregnancy. The diagnosis of abdominal pregnancy was only made possible following rupture of the amniotic sac, leading to anhydramnios, which resulted in the repositioning of the fetus to the upper maternal abdomen. This case represents the challenges faced by obstetricians in diagnosing, managing, and counselling a woman when faced with an abdominal pregnancy.

## 1. Case Report

**Figure 1:**
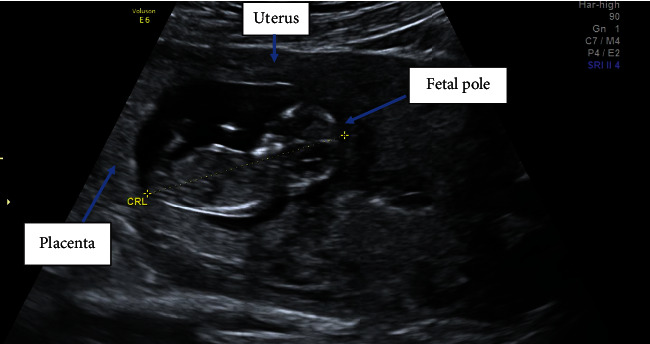
First trimester ultrasound demonstrating a viable intrauterine pregnancy.

**Figure 2:**
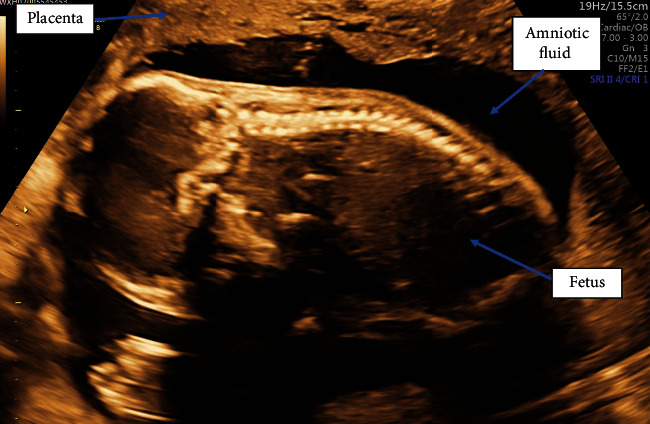
USS 20 weeks demonstrating an intrauterine pregnancy with normal amniotic fluid volume.

A 35-year-old, para 2, Afro-Caribbean woman, with no significant past medical history, presented on several occasions with abdominal pain during early pregnancy. An ultrasound (USS) scan was performed at 11 weeks gestation to exclude an ectopic pregnancy, which demonstrated a singleton viable intrauterine pregnancy with 2 small cervical fibroids (<4 cm) and a small amount of free fluid in the pouch of Douglas (Figure 1). A diagnosis of fibroid red cell degeneration was determined as an explanation for the abdominal pain. A routine anomaly USS was performed at 20 weeks which was unremarkable (Figure 2).

Due to ongoing episodes of abdominal pain, an abdominal USS was performed at 21 + 4 weeks gestation which demonstrated normal abdominal and pelvic organs and an intrauterine gestation with anhydramnios. On referral to the obstetric team, a bedside USS performed was unable to identify the fetus.

A subsequent USS was performed in the fetal medicine unit, which proved challenging due to severe oligohydramnios and uterine fibroids. The fetus could be identified and was noted to be lying laterally in the pelvis.

Initial management for preterm rupture of membranes was implemented; however, an USS 2 days later demonstrated an empty uterus with the fetus situated near the liver.

**Figure 3:**
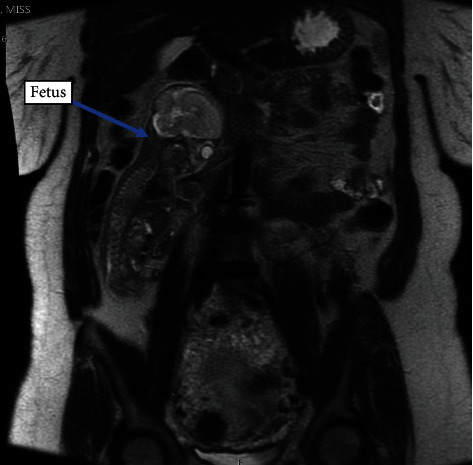
Abdominal MRI showing the fetus outside the uterus on the right side of the abdomen with the fetal head adjacent to the liver and gallbladder. The placenta is seen outside the uterus in the abdomen superior to the uterus.

The patient was referred for an urgent MRI which confirmed the diagnosis of suspected abdominal pregnancy (Figure 3).

The patient was transferred to a tertiary center for specialist multidisciplinary management at 22 + 1 weeks gestation, with access to interventional radiology services. She was extensively counselled regarding her options for conservative management or surgical termination of the pregnancy. This included discussions with the neonatal team regarding fetal outcomes at extreme prematurity as well as obstetric and anesthetic input regarding maternal risk of major hemorrhage. The patient remained committed to the pregnancy and was admitted for observation.

At 24 + 4 weeks gestation, the patient collapsed with clinical signs of acute intraabdominal bleeding. A laparotomy performed revealed 2 liters of hemoperitoneum. The placenta was embedded at the left cornua, continuous with the left fallopian tube and ovary, and adherent to the omentum.

The baby was identified extrauterine and delivered alive but in poor condition. Placental tissue continued within the myometrium; therefore, the left cornua were excised and the uterus repaired, with a blood loss of 4000 ml. Postnatally, the woman was managed in ICU and discharged on day 12. Sadly, the baby died at 24 hours of age.

## 2. Discussion

An ectopic pregnancy refers to any pregnancy that implants outside of the endometrial cavity. Abdominal pregnancy is rare, with an incidence of 1 : 10,000 to 1 : 30,000 pregnancies which constitutes 1.4% of all ectopic pregnancies [1, 2].

A primary abdominal pregnancy occurs with implantation of a fertilized ovum directly in the peritoneal cavity. More commonly, a secondary abdominal pregnancy is a consequence of rupture of a fallopian tube, ovarian, or intrauterine pregnancy resulting in subsequent peritoneal implantation [3]. Pouches surrounding the uterus are the most common sites of implantation followed by the uterine serosa and adnexa [4]. In this case, there was partial implantation into the omentum. Primary omental implantation is uncommon and associated with poor fetal outcomes and delay in ultrasound diagnosis [4].

## 3. Diagnostic Challenges

The clinical presentation of abdominal pregnancy is often heterogeneous with no pathognomic features distinguishing it from a tubal pregnancy. Consequently, the diagnosis and management of abdominal pregnancy continues to pose a challenge. It is not uncommon for an abdominal pregnancy to be diagnosed intraoperatively for a tubal pregnancy [5, 6].

Ultrasound is the modality of choice for diagnosis; however, Costa's review [7] reported that 50% of cases were missed. Diagnosis can be made challenging by the presence of fibroids, a retroverted uterus, pregnancy gestation, operator, and patient's body habitus.

Diagnosis was challenging in our case due to the presence of fibroids, anhydramnios, and false reassurance from multiple previous scans reporting an intrauterine pregnancy. The dilemma of whether this was a primary or secondary abdominal pregnancy remains as all previous USS demonstrated an intrauterine pregnancy. Her anomaly scan showed a normal volume of amniotic fluid with the placental site seen clearly at the fundus (Figure 2).

Although, on review of the 1^st^ trimester USS images, the uterus, bladder, cervix, and cul-de-sac cannot be identified in one image. Furthermore, following referral to fetal medicine, with anhydramnios, there was no free fluid in the pelvis to suggest a uterine rupture leading to a secondary abdominal pregnancy. Interestingly, the patient was hemodynamically stable with minimal abdominal pain.

Ultrasound features to aid diagnosis of abdominal pregnancy include demonstration of a fetus in a gestational sac outside the uterus or the depiction of an abdominal or pelvic mass identifiable as the uterus separate from the uterus, failure to see a uterine wall between the fetus and bladder, and recognition of close approximation of the fetus to the maternal abdominal wall and localization of the placenta outside the uterine cavity [8]. The difficulty visualizing the fetus and placenta on USS led to an MRI scan being performed which confirmed the diagnosis of abdominal pregnancy and the appearances of possible uterine rupture at the fundus.

## 4. Decision-Making Challenges

Given the significant risk to maternal health (maternal mortality 0.5-20%) [2] and the limited literature to support positive fetal outcomes, counselling patients can be challenging and additionally pose several ethical considerations. Counselling should be individually tailored and influenced by gestation, site of implantation, and maternal morbidity. Advancing gestation increases maternal risk but has the potential for encouraging fetal outcomes.

The option of surgical termination of pregnancy may be unacceptable to some patients as in our case, and therefore, significant maternal risk is accepted.

A patient's decision may also be influenced by parity as there is the potential risk of a hysterectomy. Additionally, the absence of major fetal abnormalities is likely to be a prerequisite for most patients. Previous case studies of fetal outcomes may be inaccurate due to the significant advances in neonatal care over the last decade.

## 5. Management

Once an abdominal pregnancy is suspected on USS, this should be confirmed with an MRI. Patients should be managed in a tertiary center with general surgery, vascular surgery, interventional radiology, and advanced neonatal support facilities. Cross-matched blood should be readily available in the event of emergency delivery and preparations made in anticipation of a major obstetric hemorrhage [9].

A surgical plan must be implemented in advance according to the site of implantation and its proximity to major viscera and blood vessels. Timing of delivery is also important and should be delayed if possible until fetal lung maturity is reached.

## 6. Conclusion

Abdominal pregnancy is a rare form of ectopic pregnancy with significant maternal and fetal risks. They can often be challenging to diagnose on ultrasound and therefore identified late. Individualized counselling is crucial in order to enable patients to make informed decisions regarding continuation or ending the pregnancy. Fetal and maternal outcomes depend on gestation, implantation site, and medical facilities. Advances in antenatal ultrasound and neonatal facilities are likely to improve outcomes.
